# The Emergence of a Forgotten Entity: Dermatomyositis-like Presentation of Lyme Disease in Rural Wisconsin

**DOI:** 10.7759/cureus.2608

**Published:** 2018-05-10

**Authors:** Matthew Novitch, Ahsan Wahab, Radhika Kakarala, Ridhwi Mukerji

**Affiliations:** 1 Medical Student, Medical College of Wisconsin, Wausau Wi, 54476; 2 Internal Medicine Department, Baptist Medical Center South, Montgomery, USA; 3 Internal Medicine Department, Mclaren-Flint; 4 Hospital Medicine, Aspirus Riverview Hospital

**Keywords:** lyme disease, dermatomyositis, lyme disease, borrelia, dermatomyositis

## Abstract

Dermatomyositis (DM) is one of the rare clinical manifestations of tickborne-related autoimmune presentations; we report an uncommon case of Borrelia-related DM-like presentation from rural Wisconsin. A 76-year-old female presented with fatigue, malaise, weight loss and progressive proximal muscle weakness after a flare-up of shoulder arthritis. She had a heliotrope rash and a Shawl sign in addition to generalized cutaneous erythema with edema. There was no history of tick bite, Lyme disease (LD) or DM. The physical exam revealed erythema migrans (EM) and DM-like musculocutaneous findings. Enzyme-linked immunosorbent assay and western blot were positive for LD. The patient received intravenous ceftriaxone and doxycycline for five days, leading to the resolution of EM lesions and improvement in her muscle weakness. In addition, DM-like features resolved with antiborrelial treatment and required no steroids or immunosuppressants. Workup including electromyography, skin or muscle biopsy could not be performed as the patient improved clinically. At six months post-treatment, she remained in remission.

## Introduction

Lyme disease (LD), one of the tickborne zoonoses, is a multisystem infectious disorder that involves integumentary, nervous, cardiac and musculoskeletal systems. Among pathogenic strains of *Borrelia burgdorferi *sensu lato, *Borrelia burgdorferi* is the single most common infectious etiology in the United States. Since recognition of Lyme arthritis in 1977, LD has been associated with diverse rheumatologic and dermatologic manifestations. Erythema migrans (EM), a "bullseye" type of rash that spreads close to the bite of *Ixodes* tick, is a typical sign of LD. In contrast to this, LD may induce complex dermatologic states consistent with collagen vascular disorders or autoimmune inflammatory dermatomyopathies [1-2]. We present a highly unusual case of LD from rural Wisconsin (WI). She initially presented with a flare-up of shoulder arthritis and later developed Dermatomyositis (DM)-like picture; the testing revealed a positive *B. burgdorferi* infection. Antiborrelial treatment led to the resolution of both LD and DM-like findings. To our knowledge, eight cases of *Borrelia*-related DM have been reported since 1992. This case is unique in the context of the patient’s initial presentation, clinical course, and constellation of symptoms.

## Case presentation

A 76-year-old Caucasian female presented to the emergency department (ED) with an acute exacerbation of left-sided shoulder pain. Her history was significant for chronic degenerative joint disease of shoulder and hip joints. She denied prior autoimmune disorders including rheumatologic or inflammatory myopathies. Physical exam was unremarkable and preliminary workup was negative. Myocardial infarction was ruled out and she was discharged home after intra-articular steroid injection. Over the next two weeks, she developed fatigue, malaise and progressive proximal muscle weakness. She reported a 5-pound weight loss over one-week and had mild dyspnea both at rest and exertion but no functional limitations. She denied cough, paroxysmal nocturnal dyspnea or orthopnea. She noticed upper eyelid edema and a periorbital violaceous rash, prompting her to visit a walk-in clinic. She lived in rural WI but reported no tick bites or prior LD. She had no neurological complaints and there was no evidence of EM. She was discharged with oral prednisone 20 mg daily. After few days, she returned to the ED with persistent complaints despite steroid treatment. Vitals were stable upon arrival, but she appeared lethargic. There were no signs of distress and oxygen saturation was normal on room air. The physical exam showed a positive heliotrope rash and a positive malar rash involving both nasolabial folds. She had a macular erythematous rash of extensor surface of forearms. Moreover, a widely distributed erythema of the upper neck, extending to the upper back and upper shoulders indicating a “Shawl sign” was noted. A generalized cutaneous erythema with edema involving the abdominal wall was also present. The musculoskeletal exam revealed symmetric pelvic and shoulder girdle weakness; examination of shoulder joints was unremarkable. Nervous system exam was negative for focal neurological deficits. Labs showed leukocytosis, elevated erythrocyte sedimentation rate and elevated C-reactive protein (Table 1).

**Table 1:** Laboratory findings of the patient with dermatomyositis-like features. *Normal values are given in parenthesis. Abnormal values are given in bold.

Labs	Value^*^
White blood cell count	17.8 (4.50-11.00 x 10^3^/uL) (Neutrophil 89.3%, Lymphocytes 5.8%, Monocytes 4.7%, Eosinophils 0.1%, Basophils 0.1%)
Hemoglobin	12.4(13.5-17.7 g/dL)
Hematocrit	37.5 (39.8-52.3%)
Mean corpuscular volume	91.5 (80.0-94.0 fL)
Mean corpuscular hemoglobin concentration	33.1 (33.0-37.0%)
Platelet count	311(140-440 x 10^3^/uL)
Glucose	151 (70-105 mg/dL)
Blood urea nitrogen	13.3 (7-22 mg/dL)
Creatinine	0.82 (0.50-1.50 mg/dL)
Sodium	138 (134-145 mM/L)
Potassium	4.4(3.5-5.1 mM/L)
Chloride	105(98-112 mM/L)
Bicarbonate	27(24-30 mM/L)
Anion gap	7 (6.0-14.0 mM/L)
Aspartate aminotransferase	19 (8-40 U/L)
Alanine aminotransferase	90 (7-56 U/L)
Alkaline phosphatase	230(39-117 U/L)
Total bilirubin	0.6(0.2-1.3 mg/dL)
C-reactive protein	39 (0.0-0.9 mg/dL)
Erythrocyte sedimentation rate	36 (0-30 mm/Hr)
Lactic acid	1.3(0.5-2.0 mM/L)

Alanine aminotransferase was elevated; creatine kinase was normal. Antibodies including anti-cyclic citrulline peptide antibody, antimitochondrial antibody and anticardiolipin IgA and IgM were positive; anti-JO-1 antibody was negative. Chest radiograph showed no lung pathology. Computed tomography (CT) of the chest revealed interstitial lung disease but no malignancy. Electrocardiogram showed sinus rhythm with few premature atrial complexes. The patient’s clinical presentation mimicked DM. Moreover, there was a suspicion of LD due to its increased prevalence in WI. Therefore, an enzyme-linked immunosorbent assay (ELISA) was ordered, and the patient was started on IV ceftriaxone as empiric treatment. After receiving empiric antibiotic, the patient appeared toxic, became hypotensive and developed high-grade fever. However, the worsening of cutaneous lesions was not observed. Given clinical deterioration, blood cultures were drawn, and she received fluid resuscitation. She was transferred to the intensive care unit (ICU) for close monitoring. While in ICU, two target-shaped lesions were noted on her left scapula, highly suggestive of LD-related EM; they were not previously reported or observed. The suspicion of LD became paramount leading to the addition of IV doxycycline given the patient’s deteriorating condition. After few doses of antibiotics, the patient started showing signs of clinical improvement; fever and hypotension resolved. She required no further antibiotic adjustments given her prompt response to antiborrelial treatment. She had positive ELISA for LD, later confirmed with western blot (WB). Blood cultures showed no growth. She received a total of five days treatment with IV ceftriaxone and IV doxycycline. Her DM-like presentation showed an effective response to antimicrobial treatment, and therefore, required no steroids or immunosuppressants. Her skin lesions were resolved, and muscle strength was significantly improved. Given her recovery with antiborrelial treatment, DM workup including EMG, skin biopsy (SB) or muscle biopsy (MB), was not performed. DM-like features were attributed to the LD. She was discharged home in a stable condition with oral doxycycline for two weeks. On her post-discharge follow-up, she showed no recurrent signs or symptoms of LD or DM-like clinical features. Six months after the completion of treatment, she remained in remission without recurrence of her symptoms.

## Discussion

Though the first case of LD-related myositis was reported in 1986, the association of LD with DM was first reported by Fraser et al. in 1992 [3]. Since then, there have been eight case reports of LD-associated DM involving both men and women, age ranging 31-73 years (Table 2). In our case even though there was no laboratory proven DM, there was a strong clinical picture suggestive of DM in a patient with LD. We want to highlight that unlike cases reported in the literature, LD could be a great mimicker of other autoimmune diseases like DM. To the best of our knowledge, there is no case report of DM-like clinical presentation in LD. However, there are cases of laboratory-proven DM in LD.

**Table 2:** Association of dermatomyositis with Lyme disease. CK: Creatine kinase; CPK: Creatine phosphokinase; CSF: Cerebrospinal fluid; DM: Dermatomyositis; EMG: Electromyography; ELISA: Enzyme-linked immunosorbent assay; EM: Erythema migrans; F: Female; IIF: Indirect immunofluorescence assay; LD: Lyme disease; LDH: Lactate dehydrogenase; M: Male; MB: Muscle biopsy; PCR: Polymerase chain reaction; SB: Skin biopsy; WB: Western blot; yo: year-old.

Author, Yr^†^	Age, Gender	Evidence for Lyme Disease	Presentation of Dermatomyositis	Evidence of Association (Clinical vs Lab-based)
Fraser, 1992 [3]	40-yo-M	Tick bite (+) EM lesions (+) ELISA/WB (-)	Fever, rash, weakness, Gottron’s lesions. CK (+)	Clinical (+) B. burgdorferi PCR in leucocytes (+)
Horowitz, 1994 [1]	52-yo-M	Tick bite (-) EM lesions (+) ELISA /WB (+)	Heliotrope rash, Gottron’s sign, progressive muscle weakness and pulmonary fibrosis. CPK and LDH (+), SB/MB (+)	Clinical (+)
Hoffman, 1995 [7]	73-yo-M	Tick bite (+) EM lesions (-) ELISA/WB (+)	Myalgia and proximal myopathy, heliotrope rash and epidermal atrophy of forearms. CK and aldolase (+) SB/MB (+)	Clinical (+) Borrelia PCR in SB (+) Borrelia in MB (+)
Arniaud, 1997 [4]	31-yo-M	Tick bite (+) EM lesion (+), IIF (+)	Weight loss, Gottron’s papules, periorbital edema, heliotrope rash arthromyalgia and muscle weakness. EMG (+), MB (+)	Clinical (+)
Waton, 2007 [6]	73-yo-F	Tick bites (+) EM lesion (-) ELISA and WB (+)	Heliotrope rash and muscle weakness. Aldolase and LDH (+), EMG (+), SB/ MB (+)	Clinical (+)
Nguyen, 2010 [5]	64-yo-M	Tick bite (-) EM lesion (+) Serology for LD (+)	Proximal muscle weakness associated with polyarthralgia, dyspnea and weight loss along with violaceous rash of face involving frontal, periorbital and nasolabial areas, palmar panniculitis and mechanic’s hands. ANA and Anti-JO-1 antibody (+) SB (+)	Clinical (+)
Mahfoudi, 2015 [8]	37-yo-F	Tick bite (-) EM lesion (-) Serology for LD (+)	Facial and periorbital erythema and edema along with diffuse myalgia and pelvic girdle weakness. CPK and aldolase (+)	Clinical (+)
Birlutiu, 2016 [9]	50-yo-F	Tick bite (+) EM lesion (+)	Fever, myalgia, paresthesia, weight loss and motor weakness telangiectasia and periungual coloring. CPK (+), SB (+)	Clinical (+) WB for IgG on CSF during DM (+)

Contrary to the idiopathic one, LD-associated DM shows a male predominance (five males, three females), presumably because of their increased tick exposure. LD-related cases of DM involve various intrinsic (age, gender and comorbid conditions) or extrinsic (occupation, outdoor activities, the habitation of endemic areas, non-treatment or delayed treatment) factors either for the acquisition of LD or for development of DM. Among these cases, the occurrence of DM has been associated with other autoimmune diseases such as dermatitis herpetiformis, relapsing polychondritis, idiopathic pulmonary fibrosis and cryoglobulinemia [3-6]. However, the role of these diseases in its pathogenesis remains unclear. Even though the occurrence of DM in acute LD setting is possible, most of the reported cases occurred in chronic LD infection [1, 3-7]. Perhaps, this could be due to the prolonged survival of *B. burgdorferi* in the reticuloendothelial system. To date, multiple attempts have been made to explain the development of DM in LD, but the actual mechanism remains unclear. Nonetheless, multifactorial pathogenicity is possible. Immunologically, *B. burgdorferi* can alter both cellular and humoral immunity and lead to lymphopenia, reduced suppressor cell activity, increased helper to suppressor T-cell ratio, natural killer cell abnormalities, polyclonal-B cell expansion and synthesis of oligoclonal immunoglobulins [3]. As with the idiopathic DM, autoantibodies seem to play a role in LD-related cases as well. Of note, there is a perceived inherent tendency in acquiring DM, as not all LD patients develop DM. DM, in general, is associated with HLA-DRB1*0301/DQA1*0501 in Caucasians and HLA-B27 in Asians [2]. Though the same genetic association could be valid for LD-associated DM, there are no facts that will support this notion. On the contrary, Hoffman et al. [7] speculated the role of haplotype HLA-Cw3 as the cause of predisposition for the development of *Borrelia*-related DM.

Though most of the cases are of acquired DM during LD infection, Fraser et al. [3] reported a patient with pre-existing DM who developed a flare-up after LD infection. The testing revealed a persistent *Borrelia* infection with subsequent refractory DM flare-up. The flare-up did not remit till the infection was treated. This example translates into the role of baseline autoimmunity provoked by exogenous immune stimuli in the form of infection. Apart from immune dysfunction, other proposed mechanisms include inflammation or direct tissue invasion caused by *B. burgdorferi.* This is demonstrated by the existence of spirochetes in skin or muscle biopsy specimens. Given this fact, Hoffman et al. [7] described LD-associated DM as a variant of Lyme borreliosis. In MB specimens, *Borrelia *is present in the perivascular regions and not near the damaged muscle fibers. Because of this, few authors have speculated the role of antigen-triggered response in the generation of DM [1]. Perhaps, perivascular spirochetes could release their antigens, and hence promote an antigen-triggered inflammatory response as a mechanism of surrounding muscle injury. Though it is possible that LD infection and DM might co-occur because of pure coincidence, the resolution of DM with antiborrelial treatment favors an etiological association between them. Another argument which can question this association could be the false positive LD serology in the presence of DM and not the true infectious state. However, the presence of classic LD symptoms in most of these cases will contradict this argument and support true *Borrelia* infection.

The workup of *Borrelia*-related DM is divided into three main sections, i.e., 1) testing to diagnose LD, 2) workup to ascertain DM, and 3) investigations to reveal the association of LD with DM. To our understanding, the occurrence of LD as a cause of DM might remain undetected in nonendemic regions; this is due to a small number of patients with LD and even smaller with *Borrelia*-related DM. Their detection would need high clinical suspicion and a low threshold for ordering serologic testing like ELISA or WB. Contrary to this, the serologic testing could play a crucial role while investigating a new case of DM in endemic settings; history of LD in remote or recent past might help physicians to consider this association, and therefore, to initiate LD screening. On the same note, DM cases without LD history will continue to maintain an uncertainty even in the setting of endemic infection.

Based on the literature review, an association of DM with *B. burgdorferi *infection can be established by direct or indirect means. Indirect means rely on circumstantial evidence supporting the association of DM with LD infection. Indirect evidence may include 1) positive ELISA or WB for *Borrelia* in the presence of new-onset DM, 2) development of DM during or after LD, 3) flare-up of DM triggered by LD, 4) recurrence of DM correlated with LD reinfection, 5) resolution of DM correlated with resolution of LD, and 6) resolution of DM with antiborrelial treatment.

Direct evidence involves direct visualization of spirochetes in skin or MB specimens or cultures. Though direct evidence is essential, only a few cases of LD-associated DM have been reported indicating the presence of organisms in musculocutaneous lesions. In contrast to culture requiring a special growth medium, silver staining of *Borrelia* is more convenient, yet it provides the same level of evidence. It is also to be noted that antibiotic treatment could alter the presence of *Borrelia* in SB or MB. Another limitation to the detection of spirochete in these specimens might come from a focal bacterial invasion; MB specimen, therefore, might not reveal an actual representative infection of the whole muscle tissue. Apart from culture or staining, polymerase chain reaction (PCR) of *Borrelia *DNA in SB or MB offers another direct level of evidence. However, limitations remain the same, including a scarcity of organisms and or effect of antibiotics. Furthermore, PCR might not necessarily represent viable *Borrelial* organisms.

After establishing an association of DM with LD, the treatment should focus on usual DM treatment in addition to its etiology, i.e., treatment of LD. To date, there are no clear-cut indications for the use of antiborrelial antibiotics alone or in combination with steroids or other cytotoxic therapy. In the literature, there is only one case similar to ours, when antibiotics led to the complete resolution of DM-like clinical features and required no steroids [6]. However, most of the cases needed both steroids and antibiotics for DM resolution [3, 5, 7-9]. So far, there is only one case reported by Arniaud et al. [5] who was treated with IV methylprednisolone and cyclophosphamide but not with antibiotic treatment, though Lyme serology was positive. Regarding antimicrobial therapy, Frey et al. [10] supported a four-week treatment compared to two-week treatment. Their proposal was based on the persistence of *Borrelia* PCR in three out of four patients who were treated with two-week antibiotics. Therefore, it is possible that protracted therapy might be required with persistent or residual LD cases [10].

Our case represents the classic clinical presentation of DM with negative serology. The patient lived in the LD-endemic area but reported no tick bite. She received no treatment of LD as an outpatient and came to the ED with persistent skin manifestations, myopathy and constitutional symptoms. She became critically ill while hospitalized but showed a prompt response to antiborrelial treatment and hence required no steroids or immunosuppressants, although the patient was briefly treated with outpatient prednisone.

## Conclusions

*Borrelia*-related DM-like clinical picture is one of the rare presentations of LD and hence should be considered in all patients with new-onset DM-like clinical picture in the context of living in endemic areas or history of acute or chronic LD. LD-associated DM-like clinical presentation is treatable and can be resolved completely with appropriate therapy. Testing for LD-associated DM should involve testing for LD, DM and their etiological association.
